# Accuracy of augmented reality with computed tomography-based navigation in total hip arthroplasty

**DOI:** 10.1186/s13018-023-04155-z

**Published:** 2023-09-06

**Authors:** Masahiro Hasegawa, Yohei Naito, Shine Tone, Akihiro Sudo

**Affiliations:** Department of Orthopaedic Surgery, Mie University Graduate School of Medicine, 2-174 Edobashi, Tsu, Mie 514-8507 Japan

**Keywords:** Total hip arthroplasty, Navigation, Augmented reality, Computer tomography

## Abstract

**Background:**

Augmented reality (AR) provides the surgeon with direct visualization of radiological images by overlaying them on the patient. This study aimed to evaluate the accuracy of cup placement using a computed tomography (CT)-based AR navigation system.

**Methods:**

Sixty-five prospectively enrolled patients underwent primary cementless total hip arthroplasty (THA) in a supine position using this novel AR navigation system, and changes in pelvic flexion angle (PFA) were evaluated. Absolute navigation errors were defined as the absolute differences between angles in the intraoperative navigation record and those measured on postoperative CT. Factors affecting the absolute navigation error in cup alignment were determined.

**Results:**

Mean absolute change in PFA between preoperative CT and reduction was 2.1° ± 1.6°. Mean absolute navigation errors were 2.5° ± 1.7° in radiographic inclination (RI) and 2.5° ± 2.2° in radiographic anteversion (RA). While no factors significantly affecting absolute navigation error were found for RI, absolute change in PFA between preoperative CT and reduction correlated significantly with the absolute navigation error for RA.

**Conclusion:**

This CT-based navigation system with AR enabled surgeons to place the cup more accurately than was possible by freehand placement during THA in a supine position.

## Introduction

Computer-assisted navigation has been used to achieve more precise placement of the acetabular cup in total hip arthroplasty (THA). The use of computer navigation in THA has been reported to lead to reductions in the rates of dislocation and revision [1–3]. Computer-assisted navigation systems typically display information on two-dimensional screens and require additional equipment placed outside the surgical field. Such distractions during surgical procedures might risk diverting the attention of the surgeon and could therefore impact surgical performance.

Augmented reality (AR) is a natural extension of computer-assisted surgery, providing the surgeon with three-dimensional (3D) images superimposed upon the surgeon’s view of the real world [4, 5]. AR technologies have a wide variety of applications, including direct visualization of radiological images by overlaying them on the patient and intraoperative guidance using preoperative plans [5]. The ability of AR devices to overlay virtual 3D models of soft-tissue and bone highlights how this emerging technology can improve the surgical workflow [6]. Logishetty et al. [7] demonstrated that participants using an AR headset produced smaller errors in cup orientation than those receiving guidance from the surgeon. Visually induced motion sickness has been reported with the use of AR headsets, in a phenomenon known as simulator sickness. This discomfort is characterized by nausea, disorientation, eye strain, or other oculomotor symptoms and can negatively impact the experience, acceptance, performance, and safety of the user [8].

**Fig. 1:**
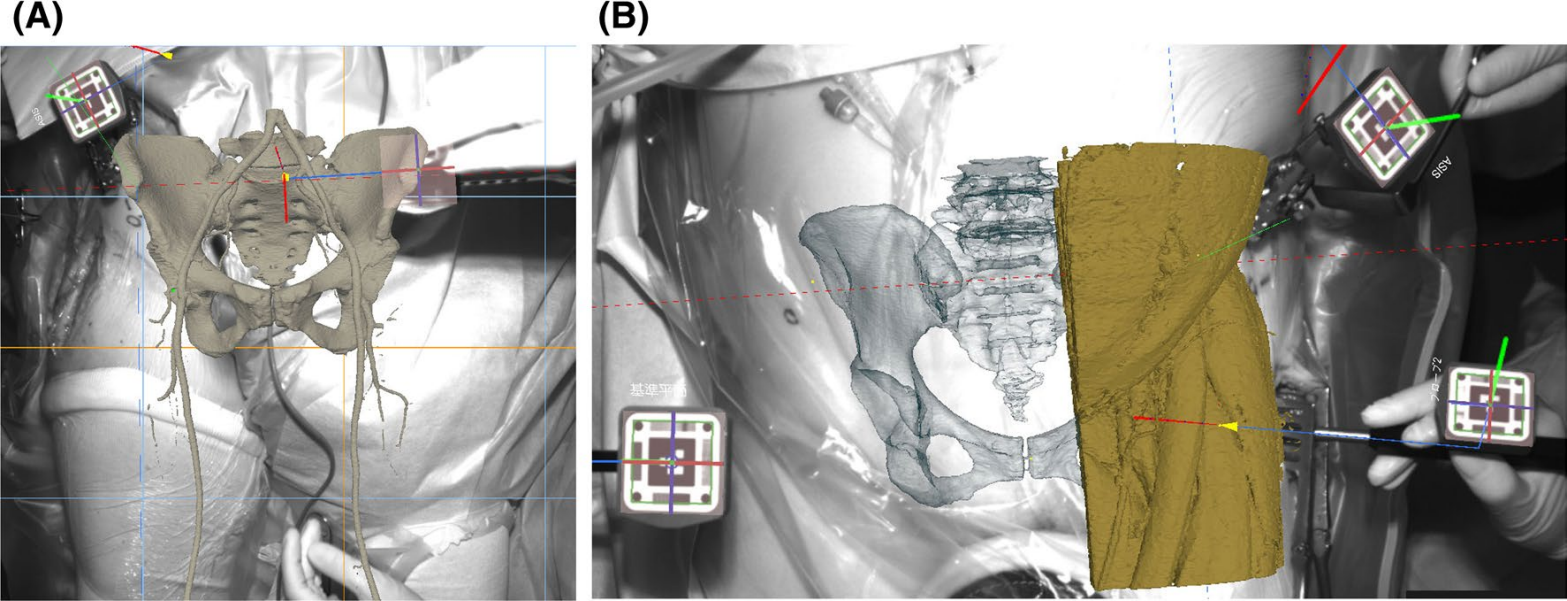
Vessels (**A**) and muscles (**B**) are depicted on the monitor. **B** Surgeons can verify the muscle depicted using the probe (yellow arrow)

To the best of our knowledge, only an AR-HIP system using a smartphone has been clinically used for AR navigation, and Ogawa et al., the developers of that system, demonstrated the benefits of AR navigation for accurate cup positioning [9, 10]. Computed tomography (CT)-based navigation with AR technology (Holonavi One Navigation System; Holonavi Medical Technology Inc., Ichinomiya, Japan) has been developed using the Unity software (Unity Technologies, San Francisco, CA, USA). The main body of this system comprises a Windows-based personal computer, infrared camera, and monitor. This system enables surgeons to see not only a 3D pelvis model but also vessels and muscles on the monitor (Fig. 1). One of the strong points of the present AR navigation is the ease of use without an AR headset. Most arthroplasty surgeons use a surgical helmet, and using an AR headset in such a situation might be difficult.

**Fig. 2:**
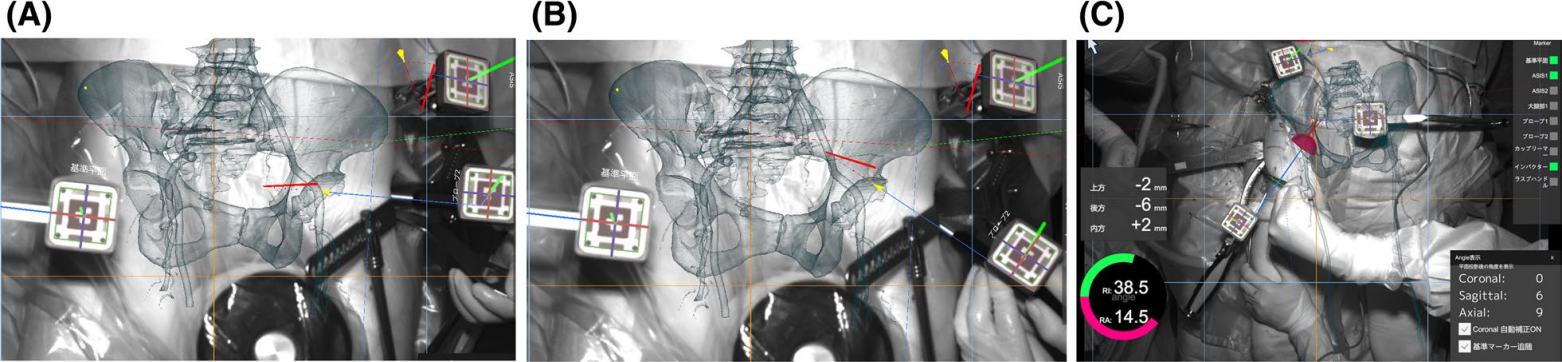
Screenshot of navigation. **A** When the anterior retractor is misplaced in a medial and caudal position, the tip of the retractor is directed to the vessel. **B** Surgeons can verify safe directions when the tip is positioned cephalad to avoid vascular injury. **C** Surgeons can view a three-dimensional model of the pelvis and cup image superimposed on the real surgical field

Using a portable navigation system with AR technology, vessels and muscles cannot be depicted on the monitor. However, using the Holonavi One, surgeons (particularly inexperienced surgeons) can easily distinguish between the tensor fascia latae, rectus femoris, and gluteus medius muscles, which might facilitate training in surgical approaches, including DAA and ALS (Fig. 1B). In cases of difficult revision, vascular injury during surgery represents one of the most devastating complications [11]. Vessels can be depicted in 3D using Holonavi One, and surgeons can easily insert screws even in difficult revisions without causing vascular injury (Fig. 1A). Surgeons need to be aware of the proximity of neurovascular structures in relation to the anterior acetabular retractor in the anterior approach [12]. The anterior retractor should be placed along the anterior acetabular rim in a cephalad direction to avoid neurovascular injury [13] (Fig. 2). Awareness of vascular structures can be improved using Holonavi One.

The purpose of this study was to evaluate the accuracy for cup placement and change in pelvic flexion angle (PFA) using this novel AR navigation system for patients undergoing THA. We hypothesized that the system would provide more accurate acetabular cup placement in THA than conventional techniques.

## Materials and methods

### Patients

Inclusion criteria were patients with symptomatic hip deformity who underwent primary THA between May 2021 and June 2022. Exclusion criteria included hips that underwent THA via a posterior approach, such as hips with high dislocation requiring subtrochanteric osteotomy and revision hip arthroplasties. Sixty-nine consecutive patients were recruited. After excluding four hips for which a posterior approach was used, a total of 65 prospectively enrolled patients underwent primary THA in a supine position under general anesthesia using the Holonavi One system. All procedures were performed by the same surgeon (M.H.). The hip was exposed via a direct anterior approach (DAA) on a traction table or with a modified Watson–Jones approach (anterolateral supine approach: ALS) with the patient in a supine position. The anterior joint capsule and iliofemoral ligament were preserved in all cases. Thirty-six hips were treated using the DAA, with the remaining 29 hips treated using the ALS. When a traction table was available, the surgeon selected the DAA. The ALS was selected in cases of severe deformity (Crowe groups 2 and 3) or excessive anteversion (> 35°) of the femoral neck. A G7 PPS Finned BoneMaster Limited Hole Shell (Zimmer Biomet, Warsaw, IN) or Squrum TT Shell (Kyocera, Kyoto, Japan) was used. CT from the pelvis to the knee joint was performed before surgery, and a preoperative plan was made using a 3D digital templating system (ZedHip, Lexi Co., Tokyo, Japan). Cup orientation was planned at a radiographic inclination (RI) of 40° and radiographic anteversion (RA) of 15° relative to the functional pelvic plane (FPP). The FPP was defined as the reference plane where the anterior pelvic plane was rotated about the inter-anterior superior iliac spine (ASIS) axis until the superoinferior axis was parallel to the table plane in the supine position as measured on preoperative CT [14, 15]. The file for the preoperative plan made using ZedHip (Lexi Co.) was transferred to the navigation system by USB.

### Surgical technique

**Fig. 3:**
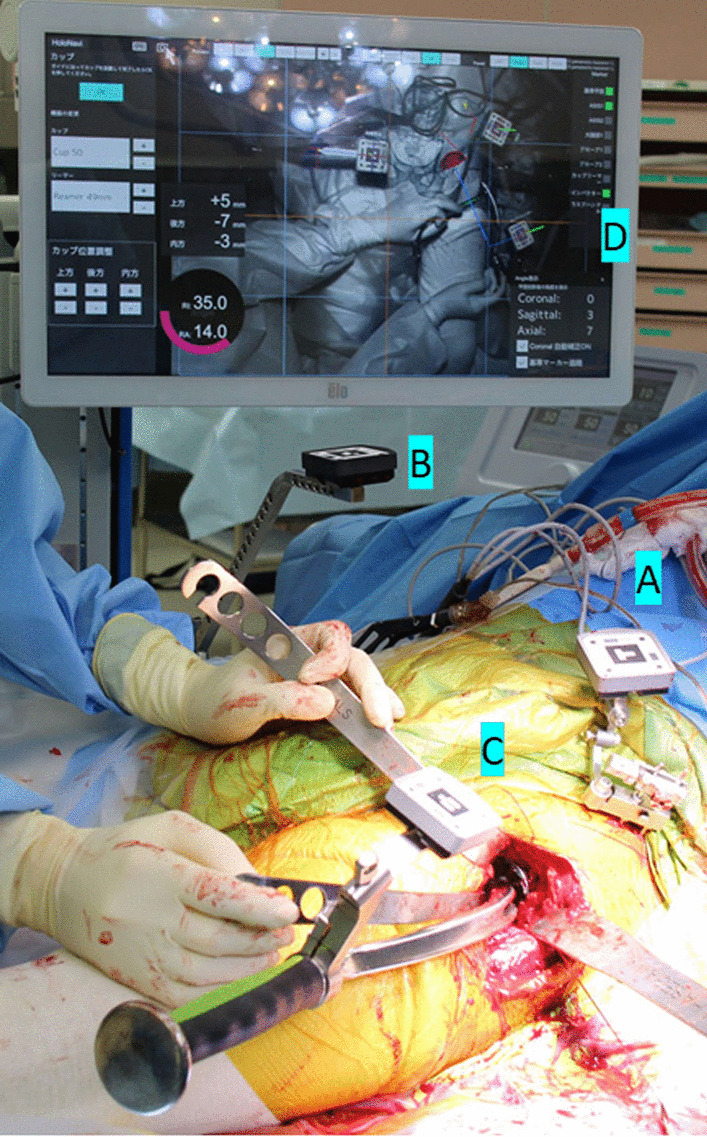
Photograph during surgery. AR markers (**A**–**C**) and the additional monitor (**D**) are demonstrated. **A** A marker is fixed to the pelvis. **B** A marker is fixed to the operating table to monitor pelvic movement. **C** A marker is attached to the cup holder

The Holonavi One can be used with patients in either the supine or lateral decubitus position. Two screws (diameter, 3.2 mm) were inserted into the iliac crest through stab incisions after draping. AR markers were firmly connected to the screws with clamps. Another AR marker was placed above the pelvis, and clamped to the rail of the operation table as a reference for PFA. The AR marker for measuring PFA had two parts. One was attached to the operation table before draping, and the other was connected to the sterile portion after draping. Registration was performed after draping. After registering bilateral ASISs and the pubic tubercle, the Holonavi One allows surgeons to view 3D models of the pelvis on the real surgical field on the monitor. The Holonavi One has an additional monitor, on which all data are displayed (Fig. 3). No head-mounted or head-up displays were used. Initial paired point matching was performed by registering three points in the posterior, medial, and superior areas of the acetabulum. These points were determined in preoperative planning to adjust CT matching. Surface matching was then performed by digitizing 30 points in the acetabulum. Standard THA instruments were used; the only modification was that an AR marker was attached to the standard reamer and standard cup holder. In patients without a history of drug allergy, bronchial asthma, severe thyroid disease, or severe renal dysfunction, preoperative contrast-enhanced CT could be performed. Preoperative contrast-enhanced CT was performed in 39 patients with informed consent. The addition of information from contrast-enhanced CT allowed vessel locations to be displayed during surgery.

After registration, direction and position of the tip of the retractor could be checked using a probe (Fig. 2). Acetabular reamer and cup images were displayed on the 3D model (Fig. 2C). When the pelvis moved coronally, sagittally, or axially during the operation, the 3D model of the pelvis moved simultaneously on the 2D monitor. Surgeons could find both the pelvis and cup, and the position of the cup and acetabular coverage could be checked on the 2D monitor. The cup was placed under navigational assistance. RI and RA were shown on the monitor. AR technology offers some advantages. By superimposing 3D images on the monitor, the surgeon can implant the cup accurately while viewing the monitor. In addition, AR technology can overlay soft-tissue images. Surgeons cannot check these views using existing CT-based navigation. Press-fit fixation was obtained in all cases after 1-mm under-reaming without the use of screws. A cementless femoral stem was implanted without navigation in all hips.

### Evaluations

PFA was defined as the angle between the anterior pelvic plane and the horizontal plane in the supine position [16, 17]. The initial PFA was determined at preoperative CT. PFA was recorded at preoperative CT, cup placement, and reduction of the hip after stem insertion. Absolute values of changes in PFA between preoperative CT and cup insertion, and between preoperative CT and reduction, were then evaluated.

CT was performed from the pelvis to the knee joint at 2 weeks postoperatively. Component positions were measured postoperatively using a 3D digital templating system (ZedHip). Cup inclination and anteversion angles were measured with respect to the FPP by one observer (Y.N.). Intra- and inter-observer reliabilities for this measurement have been examined previously [14]. Absolute target errors in RI and RA with respect to the FPP were defined as differences between preoperative target angles and angles measured on postoperative CT. Intraoperative inclination and anteversion angles using navigation were recorded. Absolute navigation errors in RI and RA were defined as the absolute differences between angles in the intraoperative navigation record and those measured on postoperative CT [14]. The percentages of hips with navigation errors over 5° and 10° were determined. The percentages of hips inside the safe zone (inclination 30°–50°, anteversion 15°–35°) were calculated as detailed by Lewinnek et al. [18]. The X-axis (transverse axis) connected bilateral ASISs. The *Z*-axis (longitudinal axis) was perpendicular to the *X*-axis parallel to the FPP. The *Y*-axis (sagittal axis) was perpendicular to the *Z*-axis on the sagittal view. Cup position was indicated using these *X*, *Y* and *Z* parameters. The accuracy of the cup position was measured using intraoperative navigation data and postoperative CT. All patients were followed after THA for a minimum of 12 months (mean 16.7 months, range 12–26 months), and complications including dislocation were examined.

### Control

As a control group, 42 previously reported patients who had undergone THA via an ALS approach in the supine position between June 2015 and June 2017 were included [14]. The target angle for RI was 40° relative to the horizontal line defined by bilateral ASISs; however, RA was targeted at 20° relative to the operation table, because we used a mechanical guide for 20° radiographic anteversion [14].

This study was approved by the Institutional Review Board at our institution (approval no. H2018-083). All patients provided written, informed consent to participate. The study was carried out in accordance with the Declaration of Helsinki.

### Statistical analysis

A power analysis was performed using G*Power 3.1.9 (Heinrich Heine Universität Düsseldorf, Germany) [19]. From a previous navigation study [20], difference (mean ± standard deviation) in CT-based navigation and conventional groups for cup inclination and anteversion was 1.6° ± 0.7° and 3.0° ± 2.8°, respectively. Based on this finding, a sample size of 14 hips in each group was considered necessary to detect a significant difference between groups (*ɑ* = 0.05, power = 0.8).

The demographic characteristics of patients, including age and body mass index, were compared between groups using a Mann–Whitney *U*-test. A chi-squared test and Fisher’s exact test were used to compare sex, diagnosis, and approach. Operation time, bleeding volume, and absolute errors in RI and RA were compared between groups using the Mann–Whitney *U*-test. Factors that affected the absolute value of navigation error in cup alignment were determined. Correlation analyses were performed using Spearman’s rank correlation test. In these analyses, dependent variables included age, body mass index, absolute PFA at preoperative CT, absolute change in PFA between preoperative CT and cup insertion, and absolute change in PFA between preoperative CT and reduction. The level of statistical significance was set at *p* < 0.05 using EZR version 1.61 [21].

## Results

Mean preoperative PFA was 3.1° ± 5.6° (range, − 16.5° to 13.5°). Mean absolute change in PFA between preoperative CT and cup insertion was 2.6° ± 1.9° (range, 0°–9°). Mean absolute change in PFA between preoperative CT and reduction was 2.1° ± 1.6° (range, 0°–8°).

**Fig. 4:**
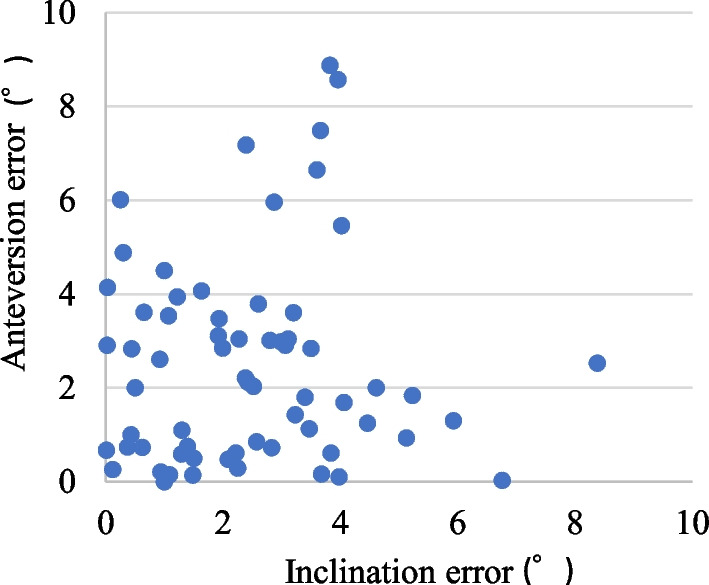
Scatter plot of absolute navigation errors in radiographic inclination and anteversion

Intra- and inter-observer reliabilities for inclination were 0.915 and 0.951, respectively, and those for anteversion were 0.963 and 0.937, respectively [9]. Mean postoperative RI and RA were 37.8° ± 2.8° (range, 30.1°–45.1°) and 16.6° ± 3.3° (range, 9.5°–23.6°), respectively. Mean absolute target errors in RI and RA (target vs. postoperative CT) were 2.8° ± 2.2° (range, 0.0°–9.9°) and 2.9° ± 2.3° (range, 0.0°–8.6°), respectively (Table 1). Mean absolute navigation errors (intraoperative navigation vs postoperative CT) were 2.5° ± 1.7° (range, 0.0°–8.4°) in inclination and 2.5° ± 2.2° (range, 0.0°–8.9°) in anteversion (Fig. 4). Mean absolute navigation errors in RI were 2.3° ± 1.6° in DAA and 2.6° ± 1.9° in ALS (p = 0.717). The mean absolute navigation errors in RA were 2.3° ± 2.4° in DAA and 2.7° ± 1.9° in ALS (*p* = 0.185). No differences in the accuracy of cup placement were found between hips treated via DAA and ALS.

**Table 1:** Absolute values of errors of the measured postoperative angles from the target angles

	AR Navigation group	Control group	*P* value
Inclination (°)	2.8 ± 2.2	6.6 ± 4.4	< 0.001
Anteversion (°)	2.9 ± 2.3	5.9 ± 4.0	< 0.001

Values are given as means ± standard deviation

Demographic characteristics of patients are shown in Table 2. No significant differences in demographic characteristics were identified between the Control and AR navigation groups except in approach and operation time (Table 2). In the Control group, mean RI and RA were 34.6° ± 5.8° and 21.4° ± 7.0°, respectively [14]. The AR navigation group showed better mean absolute error for both RI (*p* < 0.001) and RA (*p* < 0.001, Table 1).

**Table 2:** Demographic characteristics of patients

		AR Navigation group	Control group	*P* value
Age*	(years)	65.6 ± 9.3	65.9 ± 11.0	0.791
Sex	Male	10	9	0.447
	Female	55	33	
Body mass index*	(kg/m^2^)	23.9 ± 4.4	24.0 ± 3.8	0.997
Diagnosis	Osteoarthritis	64	38	0.076
	Primary	2	0	
	Secondary**	62	38	
	ONFH	1	4	
Approach	ALS	29	42	< 0.001
	DAA	36	0	
Operation time*	(min)	121.6 ± 21.0	112.2 ± 26.1	0.021
Bleeding volume*	(ml)	374.6 ± 148.8	403.6 ± 205.2	0.723

*AR* augmented reality, *ONFH* osteonecrosis of the femoral head, *ALS* anterolateral supine approach, *DAA* direct anterior approach

*Values are given as mean ± standard deviation

**Osteoarthritis secondary to developmental dysplasia of the hip

**Fig. 5:**
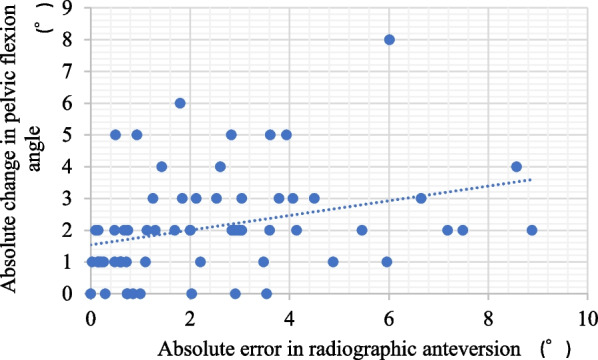
Correlation between the absolute navigation error in radiographic anteversion and absolute change in pelvic flexion angle between preoperative computed tomography measurement and reduction

No factors significantly affecting the navigation error or absolute navigation error were found for RI. However, the absolute change in PFA between preoperative CT and reduction correlated significantly with absolute navigation error in RA (*r* = 0.402, *p* < 0.001, Fig. 5). No other factors affecting absolute navigation error of RA were identified.

The percentages of hips with a navigation error exceeding 5° were 8% for RI and 12% for RA. No hips showed a navigation error > 10°. All cups (100%) were inside the Lewinnek safe zone. Mean absolute errors for cup position were 3.2 ± 2.5 mm on the *X*-axis, 2.5 ± 2.1 mm on the *Y*-axis, and 3.1 ± 2.8 mm on the *Z*-axis. No complications including pain or infection arising at pin sites were seen, and no dislocations occurred in any hips.

## Discussion

The most important finding of the present study was that this CT-based navigation with AR technology enabled surgeons to perform more accurate cup placement than freehand placement.

AR has shown great benefits in experimental studies, but only portable AR navigation systems have shown any benefit in clinical studies [9, 10, 22]. To the best of our knowledge, the present study is the first clinical research to investigate the accuracy of cup placement using CT-based navigation with AR technology. In CT-based navigation, absolute navigation errors have been reported to be in the range of 1.2°–4.6° for RI, and 1.0°–4.4° for RA [20, 23–27]. Clinical use of AR navigation systems is easy, and the accuracy for cup angle is comparable to that previously reported for CT-based navigation [20, 23–27]. Using Stryker CT-based navigation, Iwana et al. [26] reported that the mean absolute error of the cup angle was 1.8° ± 1.6° for RI and 1.2° ± 1.1° for RA. Nakahara et al. [27] showed that the mean absolute error was 1.2° ± 3.3° for RI and 1.0° ± 2.4° for RA. Matsuki et al. [20] showed that the accuracy of the cup angle was 2.8° ± 2.5° for RI and 2.8° ± 1.9° for RA. The accuracy of the cup angle reported by Iwana et al. [26] and Nakahara et al. [27] was obviously superior to the present study using Holonavi One system. However, the accuracy reported by Matsuki et al. [20] was comparable to that of the present study. The accuracy of cup angle could be affected by several factors, including the qualities of hardware and software, system measurement error, and technical errors [27]. The mean absolute errors of cup position were reported to be from 1.4 to 2.1 mm using Stryker CT-based navigation (Stryker Leibinger GmbH & Co. KG, Freiberg, Germany) [20, 26]. The accuracy of cup position in the Holonavi One seemed to be inferior to these results [20, 26]. The reamer and cup holder were not registered during surgery. These may affect the accuracy of this system. Recently, the instrument can be registered during surgery. Adding AR technology to CT-based navigation did not distinctly improve the accuracy of cup placement. The only drawbacks of Holonavi One compared to other technologies may be radiation exposure [28] and the cost of CT. The addition of preoperative contrast-enhanced CT involves a greater radiation dose and increased cost.

Ogawa et al. [10] demonstrated that the mean absolute difference using CT between targeted and measured placement angles using AR portable navigation (AR-Hip, Zimmer Biomet Japan, Tokyo, Japan) were 1.9° ± 1.3° in inclination and 2.8° ± 2.2° in anteversion. This level of accuracy is similar to that obtained in the present study. However, for the system used by Ogawa et al. [10], operating lights make viewing difficult, so surgeons are sometimes required to turn off the lights. In contrast, Holonavi One can be used under operating lights, because an infrared camera is used. The camera of Holonavi One can capture images with very narrow range from 850 to 950 nm light.

The present study did not show the reduction of vascular-related complications with visualization of vascular structures compared to the Control group, because no complications were found in both groups.

Pelvic movements during THA have been reported in the sagittal, axial, and coronal planes [29]. However, previous CT-based navigation methods could not measure pelvic movement during THA. One of the advantages of the Holonavi One system is the ability to measure PFA during THA. The CT-based navigation system can track the pelvic motion three-dimensionally via tracker, and the clinical relevance of measuring PFA during THA might be limited, although change in PFA between preoperative CT and reduction affected the RA error.

This study had some limitations. First, only a small number of patients were studied. Second, the minimum follow-up was only 12 months which was very short as a clinical endpoint in an arthroplasty study. However, the aim of this study was to evaluate the accuracy of cup placement using a novel AR navigation system. Third, CT-based platforms show significant drawbacks, including larger radiation doses and increased costs compared to conventional X-ray imaging procedures. Lastly, the use of soft-tissue overlays in the AR system was not directly studied, and the safety benefits could not be demonstrated.

## Conclusion

Mean absolute target errors (target vs. postoperative CT) were 2.8° for RI and 2.9° for RA. Mean absolute navigation errors (intraoperative navigation vs postoperative CT) were 2.5° for RI and 2.5° for RA. This CT-based navigation system with AR enabled surgeons to place the cup more accurately than was possible by freehand placement during THA in a supine position, and our hypothesis was verified. The absolute change in PFA between preoperative CT and reduction affected the absolute RA error.

## Data Availability

The datasets generated during and/or analyzed during the current study are available from the corresponding author on reasonable request.

## Abbreviations

AR: Augmented reality
CT: Computed tomography
THA: Total hip arthroplasty
PFA: Pelvic flexion angle
RI: Radiographic inclination
RA: Radiographic anteversion
3D: Three-dimensional
DAA: Direct anterior approach
ALS: Anterolateral supine approach
FPP: Functional pelvic plane
ASIS: Anterior superior iliac spine
